# Metabolic changes during carbon monoxide poisoning: An experimental study

**DOI:** 10.1111/jcmm.16522

**Published:** 2021-05-05

**Authors:** Carsten Simonsen, Sigriður Olga Magnusdottir, Jan Jesper Andreasen, Reinhard Wimmer, Bodil Steen Rasmussen, Benedict Kjærgaard, Raluca Georgiana Maltesen

**Affiliations:** ^1^ Department of Cardiothoracic Surgery Aalborg University Hospital Aalborg Denmark; ^2^ Department of Clinical Medicine Aalborg University Aalborg Denmark; ^3^ Biomedical Research Laboratory Aalborg University Hospital Aalborg Denmark; ^4^ Department of Chemistry and Bioscience Aalborg University Aalborg Denmark; ^5^ Department of Anaesthesiology and Intensive Care Pulmonary Research Center Aalborg University Hospital Aalborg Denmark

**Keywords:** carbon monoxide, carboxyhaemoglobin, metabolites, mild and severe poisoning

## Abstract

Carbon monoxide (CO) is the leading cause of death by poisoning worldwide. The aim was to explore the effects of mild and severe poisoning on blood gas parameters and metabolites. Eleven pigs were exposed to CO intoxication and had blood collected before and during poisoning. Mild CO poisoning (carboxyhaemoglobin, COHb 35.2 ± 7.9%) was achieved at 32 ± 13 minutes, and severe poisoning (69.3 ± 10.2% COHb) at 64 ± 23 minutes from baseline (2.9 ± 0.5% COHb). Blood gas parameters and metabolites were measured on a blood gas analyser and nuclear magnetic resonance spectrometer, respectively. Unsupervised principal component, analysis of variance and Pearson's correlation tests were applied. A *P*‐value ≤ .05 was considered statistically significant. Mild poisoning resulted in a 28.4% drop in oxyhaemoglobin (OHb) and 12‐fold increase in COHb, while severe poisoning in a 65% drop in OHb and 24‐fold increase in COHb. Among others, metabolites implicated in regulation of metabolic acidosis (lactate, *P* < .0001), energy balance (pyruvate, *P* < .0001; 3‐hydroxybutyrc acid, *P* = .01), respiration (citrate, *P* = .007; succinate, *P* = .0003; fumarate, *P* < .0001), lipid metabolism (glycerol, *P* = .002; choline, *P* = .0002) and antioxidant‐oxidant balance (glutathione, *P* = .03; hypoxanthine, *P* < .0001) were altered, especially during severe poisoning. Our study adds new insights into the deranged metabolism of CO poisoning and leads the way for further investigation.

## 1 INTRODUCTION

Carbon monoxide (CO) is the leading cause of injury and death by poisoning worldwide,^1^, ^2^ with approximately 50 000 emergency admissions in the United States alone each year.^3^ Deaths from CO poisoning are usually intentional or accidentally caused.^3^ CO forms during incomplete combustion of organic materials. Typical sources of intoxication are smoke from fires, indoor charcoal burning, exhaust from combustion engines and faulty residential heating systems.^4^ Exposure is particularly insidious, as CO is a tasteless, odourless, colourless and non‐irritant gas, often undetectable by human senses.^1^ Signs and symptoms of CO toxicity include headache, nausea, dizziness, confusion, chest pain, palpitation, tachycardia, cardiac dysrhythmias, cardiac and respiratory arrest, pulmonary oedema, unconsciousness and coma.^5^ If patients survive, neurological sequelae can appear.^1^

When poisoning is suspected, measurement of blood carboxyhaemoglobin (COHb) is performed.^6^, ^7^ Normal blood COHb levels range between 1% and 3%; however, up to 10% has been detected in active smokers.^8^ Mild signs and symptoms of toxicity can be present at COHb levels ranging from 3% to 24%,^5^, ^6^ while loss of consciousness normally occurs at levels above 24%, and exposure to higher levels has been shown to be fatal.^1^, ^6^ The treatment of CO poisoning consists of O_2_ therapies. While oxygen can be administered at normal pressure, normobaric oxygen therapy or as hyperbaric oxygen therapy, current evidence shows that treatment does not necessarily influence patient outcome.^9^, ^10^ Also, although COHb is the gold diagnostic standard, it is a poor predictor of the severity of acute toxicity. For example, studies have shown that the relationship between COHb levels and the severity of clinical symptoms is not well correlated because of the time between exposure cessation and the effects of supplemental oxygen treatments prior to COHb measurements.^6^, ^7^

In order to improve evaluation of the severity of CO intoxication, focus is needed on understanding the molecular mechanisms involved in its progression.^9^, ^10^ Under physiological conditions, endogenously produced CO is involved in cellular regulation of numerous physiological systems, including brain and muscle oxygen storage and utilization, relaxation of vascular and extra‐vascular smooth muscle, modulation of synaptic neurotransmission, and anti‐inflammatory, ‐apoptotic, anti‐proliferative and anti‐thrombotic processes. During exposure to abnormal exogenous CO levels, however, these physiological mechanisms are disrupted. Inhaled CO diffuses rapidly across the alveolar‐capillary membrane and becomes excessively absorbed into blood and subsequently distributed throughout the body. The distribution of CO in the body is reflected by the binding of CO to haeme proteins (eg blood haemoglobin and tissue myoglobin). Approximately 80%‐90% of the absorbed CO binds to haemoglobin (Hb) with a 200‐fold greater affinity than that of oxygen (O_2_), forming COHb, which in turn, reduces blood oxygen‐carrying capacity and induces impaired perfusion and tissue hypoxia.^1^ Although all tissues are vulnerable to CO‐induced hypoxic injury, the brain, heart and lungs are particularly vulnerable, because of their high O_2_ demands. Some of the remaining CO binds to tissue myoglobin and neuroglobin, affecting brain and muscle oxygen storage and utilization. Although the molecular mechanisms of poisoning are not fully elucidated, evidence indicates that these events lead to derangements in ion channels, inactivation of mitochondrial enzymes, immune system activation, DNA damage and cell death.^1^, ^16^

Until now, very few studies have investigated the effect of CO poisoning on the metabolome.^17^, ^18^ In a pilot metabolomics study, Ju et al^17^ investigated blood metabolites from deceased patients because of poisoning and found altered fatty acid metabolism. While this study added new insights to the deranged mechanisms of CO poisoning, it was performed post‐mortem. Hence, acute metabolite alterations reflecting ongoing toxicity may not have been reflected, because of the time elapsed from exposure cessation to sample collection. We have previously applied metabolomics to identify altered mechanisms in ischaemia‐reperfusion injury,^19^, ^20^, ^21^, ^22^ hyperoxia^23^ and the progression to acute lung injury.^20^, ^24^, ^25^ Therefore, in this current experimental study, we have been suggested that metabolomics can be used to detect metabolite changes related to the progression of acute CO poisoning. The aim was to explore the effects of mild and severe CO poisoning on arterial blood gas parameters and to associate potential metabolite alterations to increasing COHb levels. Because of the anatomical and immunological similarities between the porcine and human models,^26^ pigs were used in this study and arterial blood samples were collected before and during exposure to exogenous CO.

## 2 MATERIALS AND METHODS

### 2.1 Ethics regarding use of research animals

These experiments were approved by the Danish Animal Experiments Inspectorate (J.nr 2016‐15‐0201‐01064) and were carried out in accordance with research animal legislation. The experiments were performed at the Biomedical Research Laboratory at Aalborg University Hospital under the supervision of a veterinarian. At the end of the study, the pigs were sacrificed by an overdose of Pentobarbital.

### 2.2 Experimental animals and instrumentation

A total of eleven Danish Landrace female pigs (mean ± standard deviation, SD; 48.4 ± 2.46 kg) were included in this study. The animals were initially anaesthetized using Zoletil (Tiletamine/Zolazepam) and once intravenous access was established, anaesthesia was continued with an intravenous infusion of Fentanyl and Propofol. Intubation was performed by insertion of a 6.5 mm tube. The ventilator (Dameca DREAM) was set to a tidal volume equivalent of 8 mL/kg bodyweight, positive end‐expiratory pressure of 5 cm H_2_O and fraction of inspired oxygen (FiO_2_) to the lowest value needed to ensure an arterial partial pressure of oxygen (PaO_2_) in the normal range of 9.6‐13.7 kPa. Ventilation was set at a rate ensuring normal end‐tidal CO_2_ (ETCO_2_) values between 4.7 and 6.0 kPa. Published guidelines for fluid therapy were used.^26^, ^27^ The pigs' vital signs were monitored during the experiment; urine production and temperature with a thermo‐catheter inserted into the bladder, continuous electrocardiography (Zoll Pro Pac MD, ZOLL Medical Corporation), and arterial blood pressure through an arterial catheter inserted into the carotid artery. Arterial blood gas values along with glucose, lactate, pH, COHb and OHb were recorded frequently (ABL825 FLEX Series, Radiometer Medical).

**FIGURE 1:**
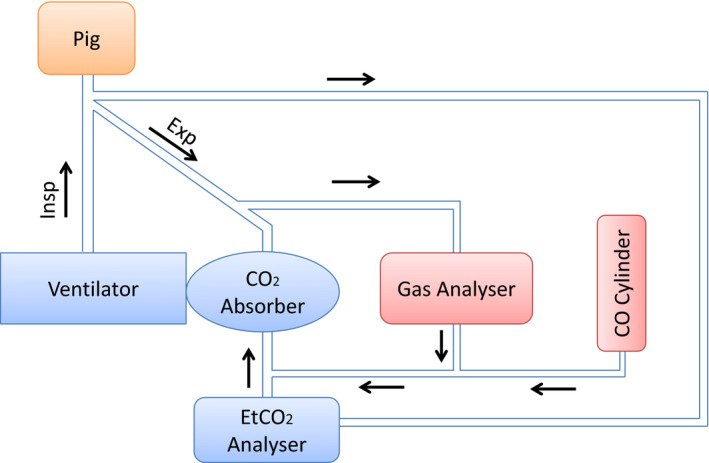
Experimental diagram showing the flow of air and CO in the experimental setup (arrows). CO, carbon monoxide; Insp, inspiration; Exp, expiration; EtCO_2_, end‐tidal CO_2_

The ventilator was connected to the pig together with a pressure cylinder containing pure CO and a closed loop connected to the CO‐monitor (Exhaust Emission Gas Analyser, Model SV‐5Q). CO was delivered with a pressure reduction valve and connected to the CO‐monitor and via the ventilator to the pig, forming a closed system to avoid leakage of CO into the laboratory room. When administering CO, the valve was opened intermittently, to avoid overdosing and to keep the maximum inhaled concentration around 1%‐2%. Constant CO‐monitoring with an alarm was used in the room to ensure safety of the laboratory personnel. The experimental setup is shown in Figure 1.

### 2.3 Experimental protocol

Blood samples for metabolite measurements were drawn at baseline and during exposure to CO, but precisely 10 minutes after inhalation of CO. The delay was to allow dispersion of CO throughout the body until COHb values indicated mild poisoning, defined as ~30% COHb, and severe poisoning, defined as >60% COHb and the point of cardiac failure. Cardiac failure was defined as a 50% decrease in cardiac output compared to baseline. This was measured using ultrasonography. Blood was collected in sterile BD Vacutainer^®^ tubes of 10 mL containing clot activator factor. Samples were left at room temperature for 30 minutes and centrifuged at 2000 *g* for 10 minutes and 4°C. Serum was obtained and stored at −80°C until analysed.

Samples were thawed for 30 minutes at 4°C and centrifuged for 5 minutes at 4°C and 12 100 *g*. Aliquots of 400 μL of supernatant were mixed with 200 μL D_2_O phosphate buffer (0.2 mol/L Na_2_HPO_4_/NaH_2_PO_4_, pH = 7.4, in 99% ^2^H_2_O). Throughout the whole procedure, samples were kept on ice. Samples were transferred to a 5 mm NMR tube and ^1^H‐NMR spectra were recorded at a temperature of 25°C using a T_2_‐filtered one‐dimensional Carr‐Purcell‐Meiboom‐Gill (CPMG) pulse sequence with water suppression, as previously described.^19^, ^20^, ^21^, ^25^ Spectral acquisition was controlled using the TopSpin 3.1 software (Bruker BioSpin).

### 2.4 Data analysis

Spectral processing, metabolite identification and quantification were performed as previously described.^20^, ^21^, ^25^ Multivariate data analysis was performed using the PLS Toolbox 6.5 (Eigenvector Research) in MATLAB (R2017a, MathWork) and the statistical analyses in the IBM^®^ SPSS Statistics software (v. 24, SPSS Inc). Unsupervised principal component analysis (PCA) was carried out to identify possible outliers and trends in the data. Data were visualized by plotting the scores of the first two principal components (PC1 and PC2), encompassing the largest variation. Metabolite quantification was performed in AMIX (Analysis of MIXtures v. 3.9.10, Bruker BioSpin) using the sum of all points under each peak of interest.

To determine differences between metabolite levels from samples collected at baseline and during mild and severe CO poisoning, standard biostatistical tests were applied.^19^, ^20^, ^21^, ^22^, ^23^, ^24^, ^25^ We first employed a Shapiro‐Wilk test to verify whether the data followed a normal distribution. Differences in group means were assessed by using analysis of variance (ANOVA) with Tukey's post hoc for multiple testing on logarithmically transformed data. Pearson correlation was applied to test the association of COHb levels and duration of exposure to CO with blood gas parameters and metabolites. A two‐tailed *P*‐value ≤ .05 was considered statistically significant.

Scatter and box‐plots were used to visualize molecular changes from before and during poisoning. Relative concentrations are presented using means and standard deviations (SD). The quality of NMR data was assessed by correlating levels of fresh arterial blood glucose and lactate recorded on the ABL800 to serum glucose and lactate measured by NMR.

## 3 RESULTS

### 3.1 Determination of CO poisoning

Carboxyhaemoglobin levels were measured in arterial blood collected from eleven Danish Landrace pigs at different time points before and during exposure to CO. To facilitate proper comparison, only blood gas data obtained from the samples used for metabolomics analyses are presented (Table 1). The measured COHb was 2.9 ± 0.5% at baseline, 35.2 ± 7.9% at mild poisoning and 69.3 ± 10.2% at severe poisoning. Mild CO poisoning was achieved after 32 ± 13 minutes, while severe poisoning occurred at 64 ± 23 minutes from baseline.

**TABLE 1:** Arterial blood gas measurements obtain from arterial blood collected from eleven Danish Landrace pigs before, and during mild and severe poisoning

	Baseline (COHb 2.9 ± 0.5%)		Mild (COHb 35.2 ± 7.9%)		Severe (COHb 69.3 ± 10.2%)		Baseline vs mild vs severe^a^	Mild vs baseline^b^	Severe vs baseline^b^
	Mean	SD	Mean	SD	Mean	SD	*P*‐value	*P*‐value	*P*‐value
pH	7.40	0.05	7.40	0.07	7.30	0.09	.002	1.00	.007
PaCO_2_ (kPa)	5.48	0.37	5.46	0.52	5.29	0.91	.67	.99	.69
PaO_2_ (kPa)	11.1	2.5	10.5	4.5	4.56	1.96	.000004	.78	.00001
COHb (%)	2.9	0.5	35.2	7.9	69.3	10.2	<.000001	<.000001	<.000001
OHb (%)	91.2	4.9	62.8	10.2	26.2	8.2	<.000001	.0004	<.000001
Glucose (mmol/L)	4.9	1.5	5.2	1.9	6.5	5.0	.93	.98	.92
Lactate (mmol/L)	1.4	0.4	1.7	1.0	6.3	1.7	<.000001	.25	<.000001

Note

Significance was assessed by means of ANOVA (^a^) with Tukey's post hoc test for multiple testing (^b^). A two‐tailed *P*‐value ≤ .05 was considered statistically significant.

Abbreviations: COHb, carboxyhaemoglobin; OHb, oxyhaemoglobin; PaCO_2,_ partial pressure of carbon dioxide; PaO_2,_ partial pressure of oxygen; SD, standard deviation.

**FIGURE 2:**
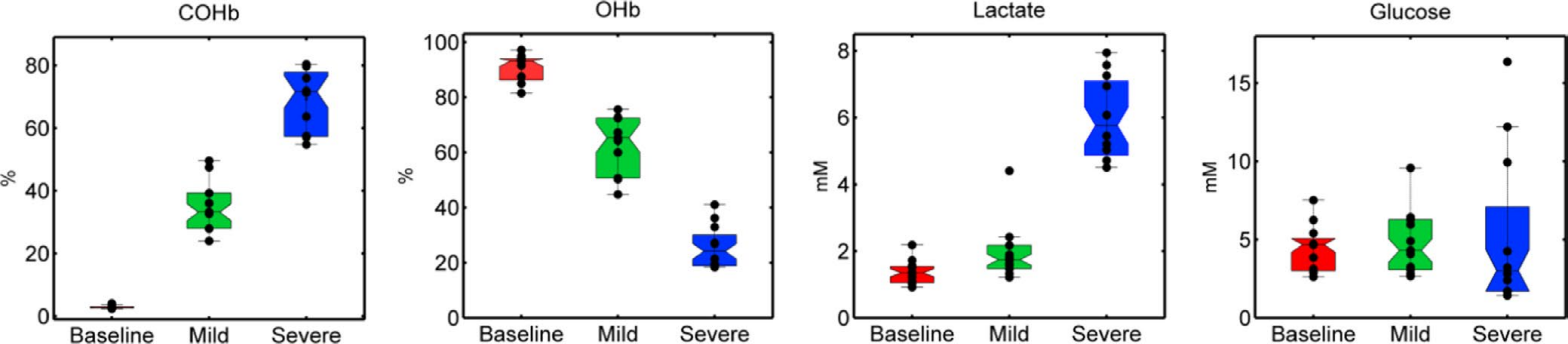
Carboxyhaemoglobin (COHb), oxyhaemoglobin (OHb), lactate and glucose values throughout the experiments. Individual measurements are represented by scores in each box‐plot indicating samples collected before exposure to CO (baseline; red) to mild (green) and severe poisoning (blue). Box‐plots explanation: data range (—), box (interquartile range), median (‐) and individual samples (●)

Increased COHb and decreased oxyhaemoglobin (OHb) levels were noticed during mild and severe poisoning (Figure 2, Table 1), indicating O_2_ displacement from Hb. PaO_2_ dropped from 11.1 ± 2.5 kPa at baseline to 4.6 ± 2 kPa during severe CO poisoning (*P* = .0001). No change was noticed in the levels of PaCO_2_ (*P* = .67). A drop in pH (*P* = .002) and an increase in lactate (*P* < .000001) levels were observed in samples obtained during CO exposure, signifying the presence of metabolic acidosis. Glucose exhibited large individual variations, especially under severe poisoning, but these changes were not statistically significant (*P* = .93). In combination, increased COHb levels, low blood oxygenation and metabolic acidosis confirmed that poisoning was successful.

### 3.2 The impact of CO poisoning on blood metabolites

**FIGURE 3:**
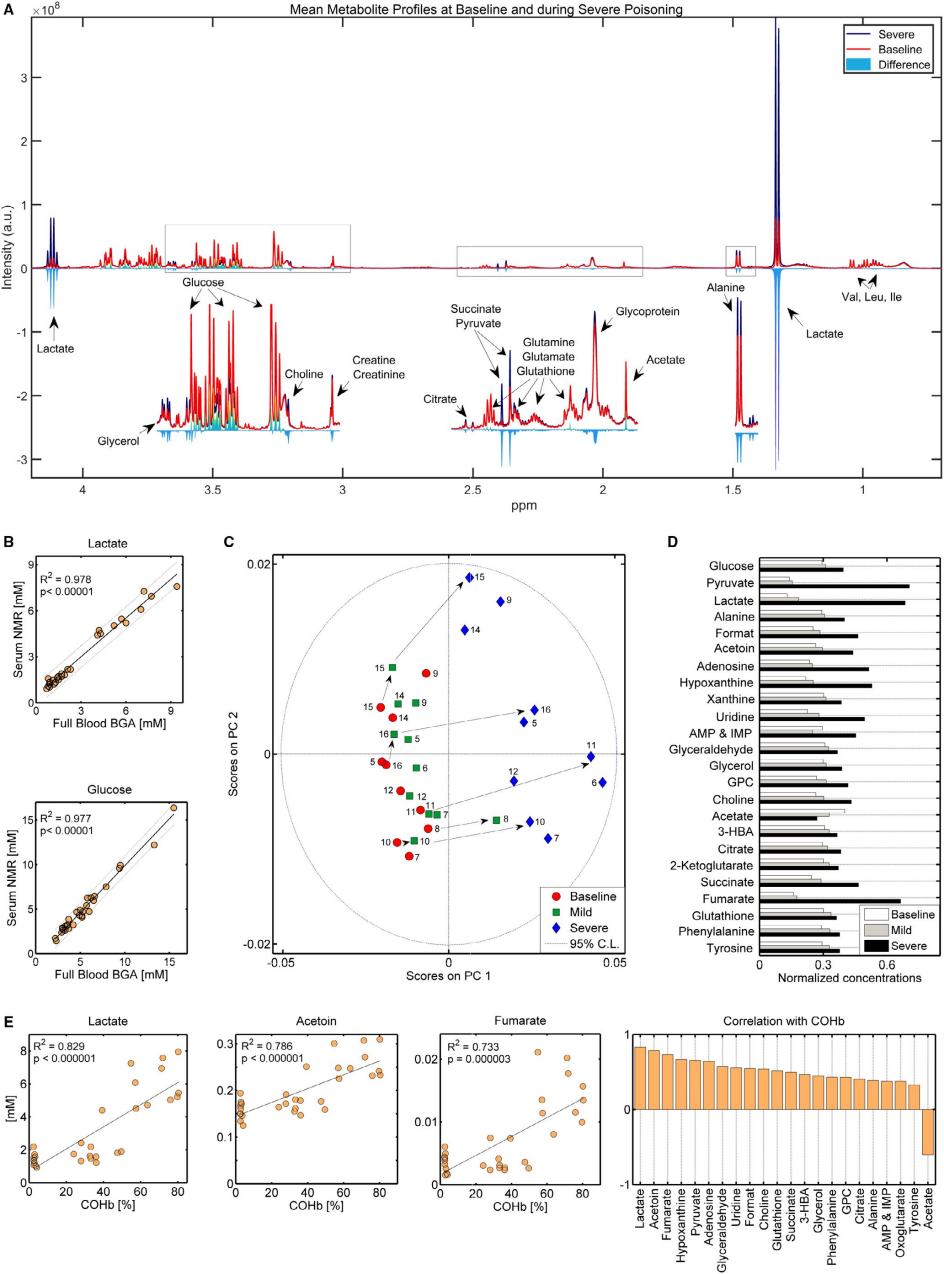
The effect of CO poisoning on metabolites. A, Overlap of mean spectra profiles from the aliphatic region showing differences between intensities of selected metabolites in samples collected before (red) and during severe poisoning (dark blue). Confirmation of CO poisoning in the Danish Landrace pig model (n = 11) by increased carboxyhaemoglobin (COHb) and decreased oxyhaemoglobin (OHb) levels. B, Data quality was checked by regressing full blood glucose and lactate values recorded on arterial blood gas analyser and serum glucose and lactate values recorded by NMR. The coefficient of determination *R*‐square shows a high degree of interrelation between these measurements, with *R* ^2^>.97. The 95% confidence intervals are shown. C, Unsupervised principal component analysis indicates shifts in blood metabolites as a consequence of increased CO. The measurements for each pig are shown to emphasize individual changes. There are two missing values, one baseline (pig no.6) and one after mild poisoning (pig no.8). D, Group mean metabolite changes as a consequence of exposure to CO. E, Metabolite correlations with COHb were calculated by means of Pearson's correlation analysis on log‐transformed data. A positive correlation indicates increases in metabolite levels, while a negative correlation indicates decreasing metabolite levels with increasing COHb

Increasing CO levels not only affected lactate and blood gas parameters, but also the composition of circulating metabolites. As depicted in Figure 3A, several metabolite intensities changed upon poisoning. Lactate, alanine, succinate, pyruvate and glycerol signals increased, while acetate decreased, with lactate undergoing the most significant alterations.

Because lactate and glucose were measured by both arterial blood gas analyser (ABL) and NMR techniques, metabolomics data quality was assessed by correlating fresh blood lactate and glucose measurements recorded on the ABG to serum lactate and glucose measured by NMR spectroscopy. High degrees of correlation were found (coefficient of determination *R*‐square, *R* ^2^ = .98; Figure 3B), indicating high data quality.

**FIGURE 4:**
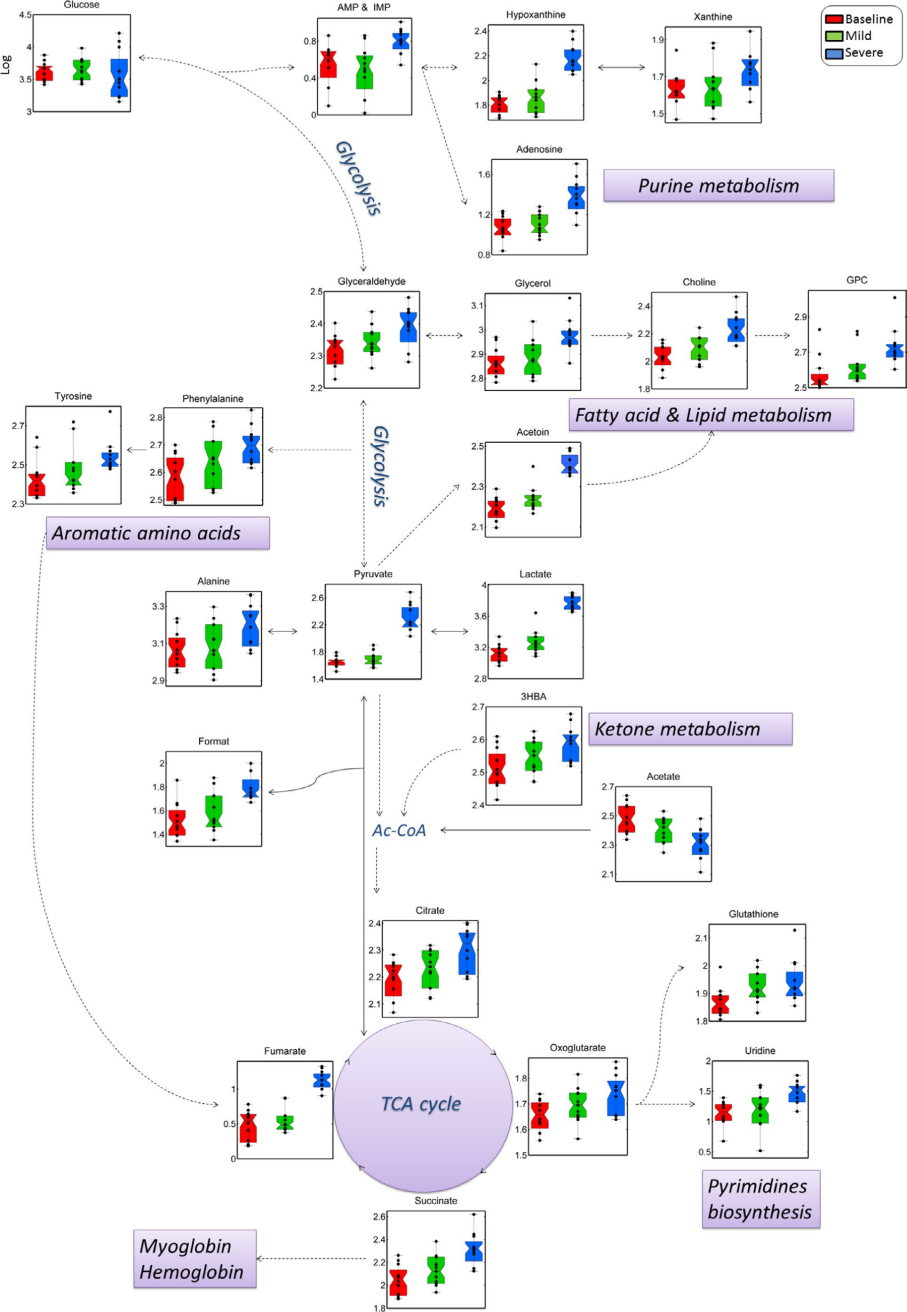
Simplified pathways showing metabolic changes as a consequence of increasing blood CO levels. Samples are visualized by scatter and box‐plots and colour coded according to baseline (red), mild CO (green) and severe poisoning (blue). The arrows represent the direction of conversion of metabolites, while the lines represent metabolite proximity (solid lines: direct conversion; dashed line: at least one metabolite is missing because of not being measured by NMR). AMP & IMP, adenosine monophosphate and inosine monophosphate; 3‐HBA, 3‐hydroxybutyric acid; Ac‐CoA, Acetyl‐CoA enzyme; GPC, glycerophosphocholine

Unsupervised pattern recognition analysis discerned between samples collected before and during exposure to CO, as well as between the degrees of poisoning (Figure 3C). While mild poisoning seemed to affect blood metabolites to a lesser extent, as reflected by the close proximity of baseline and mild samples, abnormally high COHb levels induced significant alterations, indicated by samples clustering separately along the first principal component (PC1). The most significant metabolite changes observed are presented in Table 2 and Figure 3D. Lactate, pyruvate, acetoin, aromatic amino acids (tyrosine and phenylalanine), purines (xanthine, hypoxanthine, adenosine and guanosine), tricarboxylic acid (TCA) cycle metabolites (citrate, oxoglutarate or 2‐ketoglutaric acid, succinate and fumarate), glutathione, phospholipids and glycerol were elevated, while acetate decreased with increasing blood COHb levels. Lactate, pyruvate, acetoin and fumarate were found to strongly correlate with COHb (Pearson correlation coefficient *r* ≥ .73, *P* < .0001, Figure 3E, Table 3), indicating a possible link between the degree of CO intoxication and derangements in the mechanisms involving these metabolites. One possible interpretation of altered reactions is provided in Figure 4.

**TABLE 2:** Serum metabolite changes from baseline through the mild and severe CO poisoning

	Baseline (mmol/L)		Mild (mmol/L)		Severe (mmol/L)		Baseline vs mild vs severe^a^	Baseline vs mild^b^		Baseline vs severe^b^	
	Mean	SD	Mean	SD	Mean	SD	Sig.	FC	Sig.	FC	Sig.
Lactate	1.39	0.38	1.97	0.88	6.07	1.27	0.000000000004	1.4	0.045	4.4	0.000000005
Pyruvate	0.05	0.01	0.05	0.01	0.23	0.12	0.000000000007	1.1	0.84	5.0	0.000000005
Acetoin	0.16	0.02	0.18	0.03	0.26	0.03	0.000000005	1.1	0.17	1.7	0.00000001
Fumarate	0.003	0.002	0.004	0.002	0.014	0.004	0.000000005	1.1	0.81	4.2	0.00000003
Hypoxanthine	0.07	0.01	0.08	0.02	0.16	0.05	0.00000002	1.2	0.53	2.4	0.00000006
Adenosine	0.012	0.003	0.013	0.003	0.027	0.011	0.00002	1.1	0.89	2.2	0.00005
Choline	0.11	0.02	0.13	0.03	0.18	0.05	0.0002	1.1	0.38	1.6	0.0002
Succinate	0.12	0.04	0.14	0.05	0.23	0.08	0.0003	1.2	0.45	1.9	0.0003
Formate	0.04	0.02	0.04	0.02	0.07	0.02	0.0008	1.1	0.74	1.8	0.001
Acetate	0.32	0.08	0.26	0.05	0.21	0.05	0.003	0.8	0.15	0.7	0.002
Glycerol	0.74	0.11	0.78	0.14	0.96	0.17	0.002	1.0	0.81	1.3	0.003
GPC	0.37	0.06	0.43	0.11	0.57	0.17	0.005	1.2	0.64	1.5	0.005
Citrate	0.16	0.02	0.17	0.03	0.21	0.04	0.008	1.1	0.55	1.3	0.007
Uridine	0.015	0.006	0.019	0.011	0.033	0.013	0.006	1.2	0.87	2.2	0.008
Glyceraldehyde	0.21	0.02	0.22	0.03	0.25	0.03	0.01	1.1	0.59	1.2	0.01
3‐HBA	0.33	0.05	0.35	0.04	0.40	0.05	0.02	1.1	0.44	1.2	0.01
Phenylalanine	0.37	0.07	0.42	0.09	0.48	0.09	0.02	1.1	0.34	1.3	0.01
Oxoglutarate	0.05	0.01	0.05	0.01	0.06	0.01	0.03	1.1	0.47	1.2	0.02
Glutathione	0.07	0.01	0.08	0.01	0.09	0.02	0.04	1.1	0.21	1.2	0.03
Alanine	1.21	0.30	1.27	0.37	1.65	0.45	0.03	1.0	0.96	1.4	0.05
AMP, IMP	0.003	0.001	0.002	0.001	0.004	0.001	0.01	0.8	0.82	1.5	0.05
Tyrosine	0.32	0.08	0.36	0.11	0.41	0.09	0.06	1.1	0.56	1.3	0.05
Xanthine	0.045	0.011	0.046	0.015	0.057	0.014	0.09	1.0	0.99	1.3	0.12
Glucose	4.58	1.54	4.85	2.09	5.60	5.29	0.84	1.1	0.99	1.2	0.90

Note

Significance was assessed by means of ANOVA (^a^) with Tukey' post hoc test for multiple testing (^b^). A *P*‐value *P* ≤ .05 was considered statistically significant.

Abbreviations: 3‐HBA, 3‐hydroxybutyric acid; AMP, IMP, adenosine and inosine monophosphate; GPC, glycerophosphocholine; SD, standard deviation.

**TABLE 3:** Associations between COHb and metabolites

	Correlation coefficient	*P*‐value
Lactate	0.829	<.0000001
Acetoin	0.786	<.0000001
Fumarate	0.733	.000003
Hypoxanthine	0.667	.00004
Pyruvate	0.655	.00006
Adenosine	0.642	.0001
Glyceraldehyde	0.574	.0007
Uridine	0.555	.001
Formate	0.548	.001
Choline	0.54	.002
Glutathione	0.515	.003
Succinate	0.499	.004
3‐HBA	0.466	.008
Glycerol	0.447	.01
Phenylalanine	0.431	.02
GPC	0.43	.02
Citrate	0.406	.02
Alanine	0.389	.03
AMP, IMP	0.379	.04
Oxoglutarate	0.379	.04

Note

The degree of significance was assessed by applying Pearson' correlation analysis on COHb and metabolite data. A high correlation and low two‐tailed *P*‐value (*P* ≤ .05) was considered statistically significant.

Abbreviations: 3‐HBA, 3‐hydroxybutyric acid; AMP, IMP, adenosine and inosine monophosphate; GPC, glycerophosphocholine.

## 4 DISCUSSION

To our knowledge, this is the first study to investigate metabolite responses during the progression of CO poisoning. We found that arterial blood gas parameters and several metabolites implicated in the regulation of metabolic acidosis, energy synthesis, respiration, antioxidant‐oxidant balance and lipid metabolism were altered, especially during severe CO poisoning.

Metabolites are the end products of gene, transcript and protein regulation, being strongly influenced by disease progression,^20^, ^24^ treatment interventions^19^, ^22^, ^23^ and environmental changes, including CO poisoning.^17^, ^18^ Therefore, the metabolite changes observed in this study reflect a molecular window of events occurring during exposure to exogenous CO. Although the study investigated changes in pigs, this animal model has similar physiological regulatory systems to all mammalian species, including brain and muscle oxygen storage and utilization, nitric oxide cell signalling, energy metabolism and mitochondrial respiration, steroid and drug metabolism, cellular redox balance and ROS.^28^ It is expected that some of the various modes of action differ quantitatively across species, and that some differences in dose‐response relationships between humans and animal models can be encountered because of differences in CO uptake/elimination and binding kinetics with haeme proteins.^29^ Nevertheless, results from this study can be relevant to understanding the mechanisms of action of toxic levels of CO and therefore help improve the understanding of molecular alterations in patients arriving at hospital with acute CO poisoning.

Our results indicate that exposure to CO for approximately half an hour induced mild acute poisoning, demonstrated by a 28.4% mean drop in percentage of O_2_ bound to Hb, from 91.2 ± 4.9% to 62.8 ± 7.9%, and a 12‐fold increased CO bound to Hb, from 2.9 ± 0.5% to 35.2 ± 7.9%. On the other hand, when analysing metabolites, mild poisoning had a minor impact, since most metabolites related to hypoxia (lactate, *P* = .25), energy and nutrient balance (acetate, *P* = .15), lipid peroxidation (acetoin, *P* = .17) and antioxidant‐oxidant defence (glutathione, *P* = .21) showed statistically insignificant variation. In comparison, at severe poisoning a 65% mean decrease in OHb and a 24‐fold elevated COHb were observed. Significant changes were also observed in the levels of metabolites linked to hypoxia (lactate, *P* < .0001), energy balance (pyruvate, *P* < .0001; adenosine and inosine monophosphate [AMP, IMP], *P* = .05; acetate, *P* = .002; and 3‐hydroxybutyrc acid, *P* = .01), TCA cycle (citrate, *P* = .007; succinate, *P* = .0003; oxoglutarate, *P* = .02; and fumarate, *P* < .0001), lipid metabolism (glycerol, *P* = .002; choline, *P* = .0002), antioxidant‐oxidant balance (glutathione, *P* = .03; adenosine, *P* < .0001; uridine, *P* = .008; and hypoxanthine, *P* < .0001) and other amino acids (phenylalanine, *P* = .01; tyrosine, *P* = .05).

Glucose, the key glycolytic metabolite involved in the synthesis of cellular energy, exhibited large, but statistically insignificant variation during mild and severe poisoning, compared to baseline levels. In 1985, Sokal and Kral Kowska^30^ demonstrated that glucose is neither dependent on the length of exposure to CO or severity of acute intoxication; hence, our results are in line with the literature. In contrast, metabolites downstream of glycolysis including glyceraldehyde, pyruvate, alanine and lactate were affected. Increased lactate levels with concomitant decreases in blood O_2_ and increases in COHb levels correspond with metabolic acidosis, a well‐known mechanism occurring during hypoxia caused by poisoning.^31^ Lactate has previously been found to correlate to the degree of CO poisoning^1^ and has been used as a marker of severe poisoning.^30^ In this study, we also observed a high correlation between lactate and blood COHb. Hence, our results indicate successful experimental CO intoxication, giving confidence to our findings. In addition to the glycolytic metabolites, AMP, IMP, ketones and TCA cycle metabolites were also affected, especially at severe CO poisoning, suggesting a derangement in overall energy metabolism beyond glycolysis.

In a study investigating the effect of CO on the respiratory chain, Miro et al^32^ found that mitochondrial cytochrome C‐oxidase was inhibited because of CO‐induced hypoxia. Moreover, it is well known that inhibition of the respiratory chain invokes mechanisms within the TCA cycle that slow ATP turnover and ROS accumulation.^33^ While we did not measure mitochondrial enzymes, the changes in metabolite end products of mitochondrial enzymatic activity complement the evidence mentioned above. We observed a steep increase in the levels of TCA cycle metabolites, especially during severe poisoning, including citrate, 2‐ketoglutarate, succinate and fumarate. To our knowledge, only one metabolomics study has previously demonstrated increases in blood succinate levels from deceased individuals caused by CO intoxication.^17^ While our study confirms alterations in the level of succinate, it also adds new molecular insights to the current literature. The same study further identified elevated levels of fatty acid, including myristic, palmitic, heptadecanoic, stearic, arachidonic and phosphoric acids in all deceased individuals.^17^ According to Ledoux et al,^34^ increases in blood fatty acids content are expected because of the inability of hypoxic tissue to support fatty acid beta‐oxidation. While we were unable to measure these fatty acids with NMR spectroscopy, we detected elevated cell membrane‐derived glycerol, choline and glycerophosphocholine in samples collected during poisoning. Moreover, we also found acetoin to be elevated, especially during severe poisoning, indicating a possible peroxidation of cell membranes. While lipid peroxidation is expected during CO toxicity, no study has previously identified these metabolites. In addition to lipid peroxidation, exogenous CO is known to aggravate ROS formation and promote cellular damages. Current research shows that during CO poisoning, xanthine oxidase and dehydrogenase are involved in processes that produce peroxides and superoxides.^16^ While we did not measure xanthine oxidase and dehydrogenase, their metabolite substrates, hypoxanthine and xanthine, were significantly altered during severe toxicity. Lastly, the antioxidant metabolite glutathione, involved in ROS balance, was significantly elevated during mild and severe poisoning, indicating derangements in oxidant‐antioxidant balance.

There are several limitations to our study, including the relatively low number of animals enrolled, the lack of a control group and of an independent validation study to confirm the observed molecular alterations. However, we have chosen to show each individual pig's metabolite profile to emphasize that the CO‐related changes are uniform throughout the samples. Moreover, because each pig functioned as its own control, the potentially individual variation that otherwise may add confounding to the data has been reduced. In addition, it is always challenging to translate findings from animal studies to human conditions; however, pigs are similar in physiology and anatomy to humans, and hence the results can more easily be translated to a human setting.^26^ Moreover, to the best of our knowledge, this study is the first metabolomics study of severe CO poisoning in a large animal model. We examined metabolites solely on samples obtained until severe heart failure. Measuring changes during recovery from poisoning would have provided additional information about the mechanisms involved during CO removal from metabolism. This must be a focal point in future studies, during which different treatment modalities and their effects on the return to normal conditions could also be tested. The treatment of both mild and severe CO poisoning has not evolved greatly during the last decades and still consists of oxygen therapy, which in severe cases can be administered in a hyperbaric pressure^35^ chamber. There is ongoing debate about whether oxygen therapy reduces the risk of developing neurological sequelae or mortality^8^, ^9^, ^10^, ^11^ or whether it might even be harmful, because of barotrauma, pulmonary oedema, seizures and free radical generation.^1^, ^10^ Therefore, there is still a need to understand the molecular basis of current treatments, which may combat the toxic effects of CO binding to haemoglobin, but fail to counteract the direct cellular actions of CO. In future studies, it would be interesting to explore the metabolic changes during a slightly less severe poisoning for a longer duration of time, including changes related to ongoing treatments until restoration of metabolite balance occurs.

## 5 CONCLUSION

Our experimental study adds new insights to the deranged metabolite mechanism of CO poisoning, including hypoxia, ROS formation and peroxidation. This study leads the way for further studies investigating this area. Ideally, studies looking into the effects of hyperbaric oxygen therapy versus normobaric oxygen should be considered. With further research, it may be possible to pinpoint markers that can be used to guide the intensity of oxygen therapy and thereby diminish some of the harmful side effects of oxygen therapy.

## CONFLICT OF INTEREST

The authors report no conflict of interest. The authors alone are responsible for the content and writing of this paper.

## AUTHOR CONTRIBUTIONS

**Carsten Simonsen:** Conceptualization (lead); Formal analysis (equal); Investigation (lead); Methodology (lead); Project administration (supporting); Writing‐original draft (lead); Writing‐review & editing (equal). **Sigridur Olga Magnusdottir:** Conceptualization (supporting); Formal analysis (equal); Investigation (supporting); Methodology (supporting); Supervision (supporting); Writing‐review & editing (equal). **Jan Jesper Andreasen:** Conceptualization (supporting); Investigation (supporting); Supervision (supporting); Writing‐review & editing (supporting). **Reinhard Wimmer:** Methodology (supporting); Software (supporting); Writing‐review & editing (supporting). **Bodil Steen Rasmussen:** Conceptualization (supporting); Investigation (supporting); Supervision (supporting); Writing‐review & editing (equal). **Benedict Kjærgaard:** Conceptualization (lead); Funding acquisition (lead); Investigation (equal); Methodology (equal); Project administration (lead); Resources (lead); Supervision (lead); Writing‐review & editing (equal). **Raluca Georgiana Maltesen:** Conceptualization (supporting); Data curation (lead); Formal analysis (lead); Investigation (equal); Methodology (lead); Software (lead); Supervision (supporting); Visualization (lead); Writing‐original draft (lead); Writing‐review & editing (equal).

## Data Availability

The data that support the findings of this study are available from the corresponding authors upon reasonable request.
